# Cartilage Oligomeric Matrix Protein in Idiopathic Pulmonary Fibrosis

**DOI:** 10.1371/journal.pone.0083120

**Published:** 2013-12-20

**Authors:** Louis J. Vuga, Jadranka Milosevic, Kusum Pandit, Ahmi Ben-Yehudah, Yanxia Chu, Thomas Richards, Joshua Sciurba, Michael Myerburg, Yingze Zhang, Anil V. Parwani, Kevin F. Gibson, Naftali Kaminski

**Affiliations:** 1 Dorothy P and Richard P Simmons Center for Interstitial Lung Diseases, Division of Pulmonary, Allergy and Critical Care Medicine, University of Pittsburgh, School of Medicine, Pittsburgh, Pennsylvania, United States of America; 2 Department of Pathology, University of Pittsburgh, School of Medicine, Pittsburgh, Pennsylvania, United States of America; 3 Pittsburgh Development Center, Magee-Women’s Research Institute and Foundation, University of Pittsburgh, School of Medicine, Pittsburgh, Pennsylvania, United States of America; 4 Pulmonary, Critical Care and Sleep Medicine, Yale School of Medicine, New Haven, Connecticut, United States of America; University of North Dakota, United States of America

## Abstract

Idiopathic pulmonary fibrosis (IPF) is a progressive and life threatening disease with median survival of 2.5–3 years. The IPF lung is characterized by abnormal lung remodeling, epithelial cell hyperplasia, myofibroblast foci formation, and extracellular matrix deposition. Analysis of gene expression microarray data revealed that cartilage oligomeric matrix protein (COMP), a non-collagenous extracellular matrix protein is among the most significantly up-regulated genes (Fold change 13, p-value <0.05) in IPF lungs. This finding was confirmed at the mRNA level by nCounter® expression analysis in additional 115 IPF lungs and 154 control lungs as well as at the protein level by western blot analysis. Immunohistochemical analysis revealed that COMP was expressed in dense fibrotic regions of IPF lungs and co-localized with vimentin and around pSMAD3 expressing cells. Stimulation of normal human lung fibroblasts with TGF-β1 induced an increase in COMP mRNA and protein expression. Silencing COMP in normal human lung fibroblasts significantly inhibited cell proliferation and negatively impacted the effects of TGF-β1 on COL1A1 and PAI1. COMP protein concentration measured by ELISA assay was significantly increased in serum of IPF patients compared to controls. Analysis of serum COMP concentrations in 23 patients who had prospective blood draws revealed that COMP levels increased in a time dependent fashion and correlated with declines in force vital capacity (FVC). Taken together, our results should encourage more research into the potential use of COMP as a biomarker for disease activity and TGF-β1 activity in patients with IPF. Hence, studies that explore modalities that affect COMP expression, alleviate extracellular matrix rigidity and lung restriction in IPF and interfere with the amplification of TGF-β1 signaling should be persuaded.

## Introduction

Idiopathic pulmonary fibrosis is a chronic and devastating disease without a known etiology [1]. To date, IPF remains incurable with a median survival of 2.5 to 3 years [2] and it has the worst prognosis among interstitial lung diseases [3]. The prevailing hypothesis of disease pathogenesis suggests the disease begins as an alveolar epithelial injury with aberrant alveolar re-epithelialization [4]. What is believed to follow is a cascade of events including local changes in epithelial cell phenotypes, fibroblast-myofibroblast transformation, macrophage activation, epithelial cell apoptosis, release of a variety of cytokines, chemokines, and growth factors, including transforming growth factor β1 (TGF-β1). TGF-β1 is probably the most studied among them, because of its wide known roles in extracellular matrix deposition, as well as extensive effects on fibroblast and epithelial cell phenotypes [5]–[7]. While the relative contribution of these events is unclear, the end result is extensive lung remodeling, uncontrolled extracellular matrix deposition and formation of myofibroblast foci.

We and others have applied genome scale transcript profiling techniques of human IPF lungs to better understand the disease, identify novel targets for therapeutic interventions as well as new biomarkers [8]–[13]. These studies have led to generation of expression profiles, and usually focused on one or two target molecules [8], [10], [14]–[16], but they still contain a wealth of information and should be mined for more. Recently, re-analyzing the datasets, we discovered that the cartilage oligomeric matrix protein (COMP), a protein never studied in the context of IPF, is among the top up-regulated genes in IPF lungs in published datasets [17].

Cartilage oligomeric matrix protein (COMP) is an extracellular matrix protein that is mainly localized to tendon, cartilage, and pericartilage tissues [18]. COMP has four epidermal growth factor binding domains, 8 TSP-3 repeats, and a thrombospondin C-terminal domain, which together are responsible for binding interactions with other proteins and extracellular matrix components such as TGF-β1 [19], [20]. COMP interacts with multiple matrix components, including collagens type I, II, and IX, proteoglycans, non-collagenous matrix proteins such as fibronectin and matrilins [21]–[23]. Most importantly COMP functions as matrix assembling facilitator and plays a role in the stability of the collagen network. COMP binds and brings five collagen molecules close to each other and promotes collagen fibril formation [24]. However, COMP doesn’t bind to the formed collagen fibrils; instead it works as a catalyst to arrange the collagen molecules for early and abnormal fibril formation and thus may contribute to matrix rigidity.

Increases in COMP have been reported in several diseases [25]–[27]. In rheumatoid arthritis and osteoarthritis injury to chondrocytes leads to increased secretion of COMP [25] and interaction of COMP with rheumatoid arthritis synovial fibroblasts through integrins has been reported [28], [29] COMP secretion from skin fibroblasts has been reported in affected skin of keloids [30] and systemic sclerosis patients [31]–[33]. Elevations of COMP have also been reported in vascular atherosclerosis [34], systemic lupus erythematosus (SLE) [35], renal fibrosis [36], degenerating acinar cells of chronic pancreatitis [37], and liver cirrhosis [38]. While increases in COMP have not been reported in lung fibrosis, we noticed that COMP was increased in some of our microarray datasets.

Based on these observations, we decided to investigate the role of COMP in IPF. We analyzed the expression of COMP in a larger set of lungs, localized its protein over-expression in IPF lungs and determined its regulation and effects on normal human lung fibroblasts and determined the relationship between elevated COMP serum levels and measures of disease severity in IPF.

## Materials and Methods

### Gene Expression Microarray

The IPF and control lung tissues were obtained from University of Pittsburgh Health Sciences Tissue Bank (Pittsburgh, PA). The experimental materials, procedures, samples collection, IRB, and statistical analysis have been previously described by us [17], [39]. The gene expression microarray was performed on 15 controls and 23 UIP samples. The data is available at Gene Expression Omnibus (GSE-10667).

### nCounter® Gene Expression Analysis

We extracted total RNA from 154 Control lungs and 115 IPF lung tissues obtained from the Lung Tissue Research Consortium (LTRC) for nCounter® Analysis System (Nanostring, Seattle, WA) validation of COMP mRNA expression. Details about the samples are available at the LGRC (Lung Genomics Research consortium) website (https://www.lung-genomics.org). 500 ng of total RNA were hybridized to a 3′ biotinylated capture probe and a 5′ reporter probe tagged to a fluorescent barcode. Following overnight hybridization at 65°C, the samples were transferred to the nCounter® Prep Station, excess probes were washed out, and the probe-RNA complexes were bound and immobilized on streptavidin-coated cartridges. The cartridges were scanned in the nCounter® Digital Analyzer using 1155 fields of vision. The data was normalized using GUSB as the housekeeping gene.

### Cell Culture and Transfection

Early passages (1–3) of primary normal human lung fibroblast (NHLF) (Lonza Ltd, Basel Switzerland) were cultured in a humidified atmosphere containing 5% of CO_2_ in incubator (Kendro Lab, New Town, CT) at 37°C and maintained as per the supplier’s instructions. For hypoxic conditions, cells were placed in hypoxic conditions with 1% of oxygen for 24 hours. All cells were grown until 70–80% confluence. Whenever indicated, cells were stimulated with recombinant TGF-β1 (R&D, Minneapolis, MN) and/or transfected with 50 nM siCOMP and their corresponding negative controls (Thermo Scientific Dharmacon, Lafayette, CO) using Lipofectamine 2000 (Invitrogen, Carlsbad, CA) according to the manufacturer’s instructions.

### RNA Extraction and Real-time RT-PCR Analysis

Total RNA were extracted from NHLF. All reagents (including primers for COMP) and analysis software used for qRT-PCR experiment were obtained from ABI, (Foster City, CA) and performed according to the vendor recommendation as previously described by us [16].

### Protein Isolation and Western Blot Analysis

Lung tissues and NHLF were lysed, harvested following the manufacturers’ protocol (Thermo Fisher Scientific™, Rockford, IL). The concentrations of protein were measured by using Pierce’s Bicinchoninic acid (BCA) (Pierce, Rockford, IL). For Western blot analysis, equal amounts of cellular extracts (10 µg) were separated on 10% SDS-PAGE gels and transferred to PVDF-Plus membranes (GE Osmonics, Trevose, PA). Western blots were performed with antibodies against COMP (1∶1,000; Lifespan Bioscience, Inc., Seattle, WA), β-actin (1∶10,000; Sigma – Aldrich, St. Louis, MO), P-SMAD3 (1∶1,000; Cell Signaling Technology, Inc. Beverly, MA). After incubation with the respective secondary antibodies, specific bands were visualized by autoradiography using enhanced chemiluminescence according to the manufacturer’s instructions (PerkinElmer Life Sciences, Boston, MA). Densitometry was performed using the shareware, ImageJ (http://rsbweb.nih.gov/ij/).

### Immunohistochemistry

Paraffin embedded IPF and control lungs were obtained from University of Pittsburgh Health Sciences Tissue Bank (Pittsburgh, PA). Tissue slides were deparaffinized in serials: 100%, 90%, 80%, 70%, 60% and 50% ethanol and rehydrated three times in PBS each time for 10 minutes. Slides were incubated for 45 minutes with 5% donkey serum in Tris-buffered saline (TBS) pH 7.4 containing 3% bovine serum albumin (Sigma-Aldrich) and incubated for 4 hours with primary antibody. After five washes with 0.5% BSA in TBS for 5 minutes each time, slides were incubated in a biotinylated donkey anti-rat secondary antibody for 30 minutes. After 2 washes with 0.5% BSA in TBS for 10 minutes each, the slides and arrays were then incubated with streptavidin-linked alkaline-phosphatase (Jackson Immuno Research, West Grove, PA). Slides were washed again and incubated for 15 minutes in Fast Red substrate (DakoCytomation, Carpinteria, CA) to detect the activity of alkaline-phosphatase. Briefly, tissue sections and arrays were washed for five minutes in water and counterstained using Mayer’s Hematoxylin (DakoCytomation, Carpinteria, CA). The images were visualized with Olympus™ microscope, PROVIS (Olympus America Inc., Melville, NY).

### Confocal Imaging

Frozen IPF lung slides were fixed in 2% Paraformaldehyde (Sigma-Aldrich, St. Louis, MO) for 20 minutes and permeabilized using 0.1% Triton X in PBS for 15 minutes, followed by rehydration in PBS, washes with 0.5% BSA in PBS and blocking with 5% donkey serum (Sigma) in 3% BSA in PBS for 45 minutes. Slides were incubated with anti-COMP (Accurate Chemical and scientific corporation, Westbury, NY), Vimentin (Abcam, Cambridge, MA), or pSMAD3 (Lifespan Bioscience, Inc., Seattle, WA) antibodies in a blocking solution overnight at 4°C. Secondary antibodies, nucleus staining (DAPI) and confocal imagining were performed as previously described by us [16], [40].

### Longitudinal Study Population

All patients were evaluated at the University of Pittsburgh Medical Center, Pittsburgh, PA and studies were approved by the Institutional Review Board (IRB) at the University of Pittsburgh. The diagnosis of IPF was established on the basis of American Thoracic Society (ATS) and European Respiratory Society (ERS) Criteria [41] and surgical lung biopsy when clinically indicated. Clinical data were available through the Simmons Center Database at the University of Pittsburgh. Smoking status was defined as previously described [42]. All patients signed informed consent to participate in the study. Subjects enrolled in the study were followed at intervals of 3 to 4 months according to usual care practices at the Dorothy P and Richard P Simmons Center for Interstitial Lung Diseases. Physiologic data (Pulmonary Function Tests [PFT] and oxygen desaturation studies) and physician assessments were performed at all visits. Radiographic studies (X-rays or Computed Tomography Scans) were performed when clinically indicated and blood samples were collected and pulmonary function tests (PFT) were examined in intervals of 3 to 4 months. The demographic and the clinical information of the patients in the longitudinal study are shown in the Table 1.

**Table 1:** Demographic and the clinical information of the patients in the longitudinal study.

Variables	Characteristics	IPF (N = 23)
Gender	Male	16
	Female	7
Race	Caucasian	23
Smoking	Smokers	15
	Non smokers	8
Age	Mean±SD	68.1±8.6
	Male	67.8±8.1
	Female	68.9±10.4
Baseline PFTs	FVC%(predicted)	68.6±17.9
	DLCO%(predicted)	48.3±16.5
	CPI	50.7±11.4

FVC: Force Vital Capacity, DLCO: Diffusing Capacity of Lung for Carbon Monoxide,

CPI: Composite Physiological Index.

### Enzyme-Linked Immunosorbent Assay (ELISA)

The IPF patients were recruited in longitudinal study as described in “Study Population”. Participants were followed up for 2.5 years while their blood was drawn and PFT were examined. We measured COMP level in serum of IPF patients and controls using COMP ELISA kit (AnnaMar Medical AB, Goeteborg, Sweden). The experimental procedures were followed as recommended by the vendor and data were analyzed by utilizing Delta soft 111 version 2.243 (Bio-Rad, Hercules, CA).

### Statistical Analysis of Data

All values were presented as mean ± SD. Group comparisons were made using an unpaired, two-tailed Student’s t-test for normally distributed data. A level of p<0.05 was considered statistically significant. Longitudinal study of COMP and all the PFT for 23 patients within 120 days of any blood draw were used for correlation analysis of COMP level to FVC %. A data set with one record per PFT occasion, associating each PFT with COMP level from the blood draw nearest it, was generated for all PFT of 23 patients within 120 days of any blood draw. A generalized estimating equation was used to fit to the data, based on the reference of Yan et al [43] with FVC % predicted as dependent and COMP as independent variable, and accounting for the subject effect by assuming an exchangeable within-subject correlation structure.

## Results

### COMP Gene and Protein Expression is Higher in IPF Lungs

**Figure 1:**
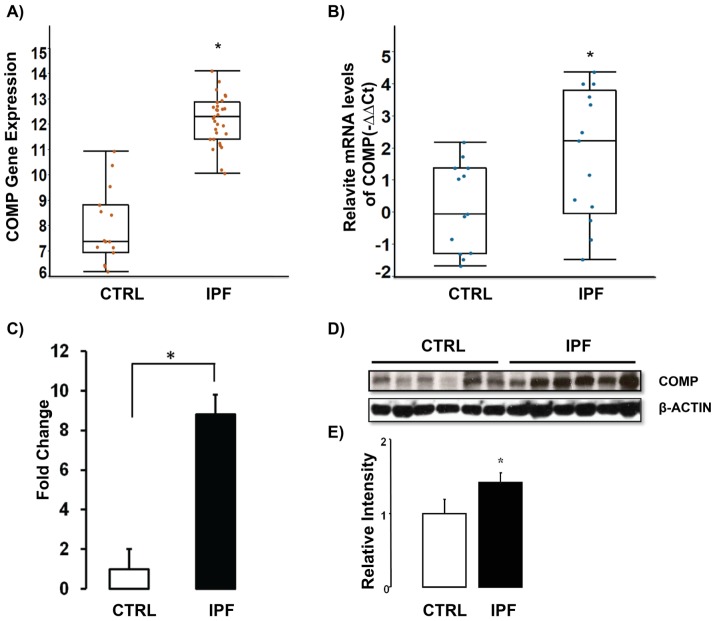
COMP gene and protein levels are increased in IPF lungs. (A) Microarrays analysis revealed an increased COMP gene expression in 23 IPF lungs compared to 15 control lungs (A) and verified in 13 IPF lungs and 13 control lungs by using qRT-PCR (B). (C) COMP mRNA levels were determined by nCounter® in 115 IPF lungs and 154 control lungs. (D–E) Protein levels of COMP were determined in IPF lungs (n = 6) and control lungs (n = 6) by using western blot and quantified by using ImageJ (p<0.05). β- Actin was used as loading control and the western blot shown is a representative of three repeated experiments.

**Figure 2:**
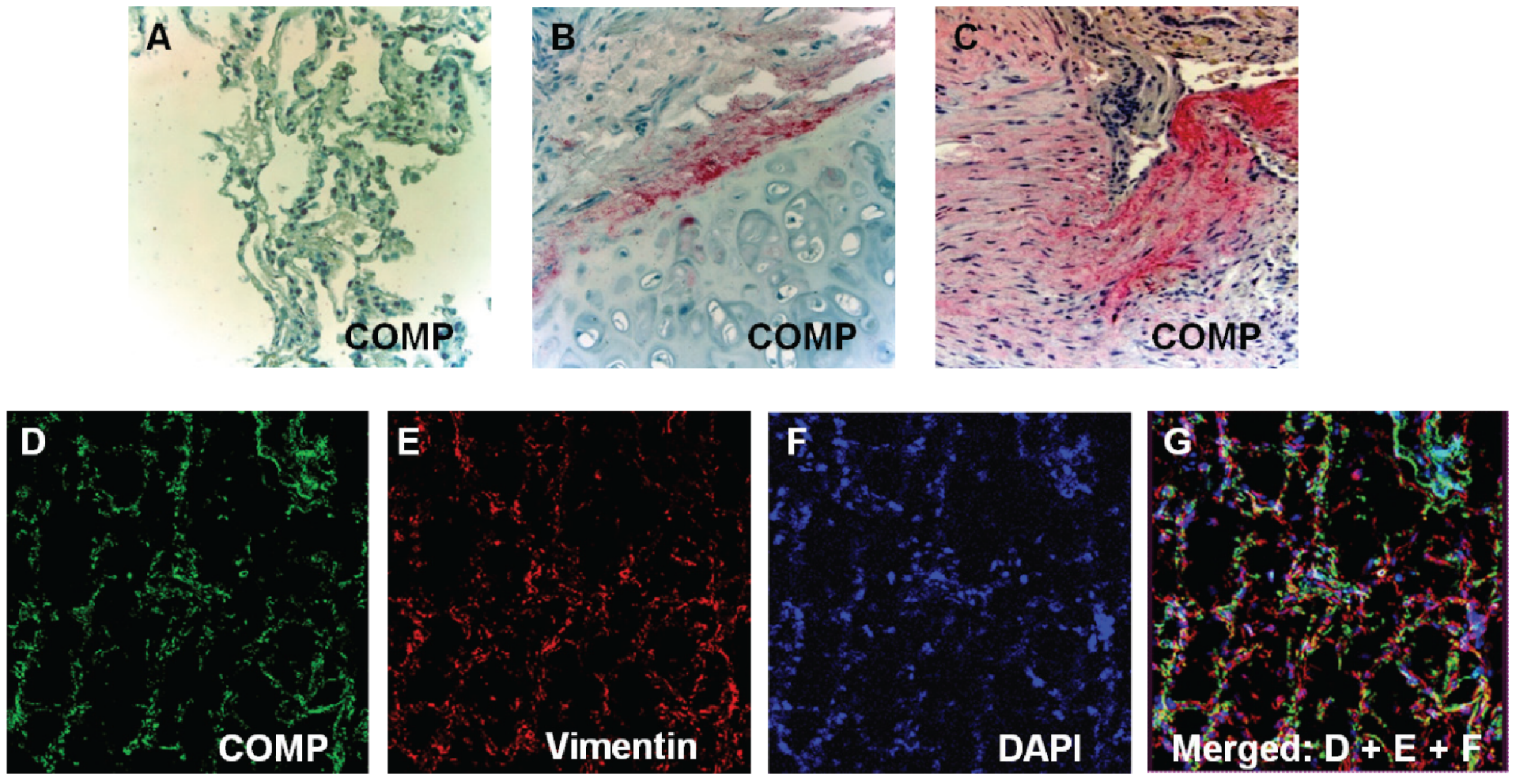
COMP is localized in fibrotic regions of idiopathic pulmonary fibrosis lungs. **(A–B)** Localization of COMP protein (red) in tissue obtained from control lung: a healthy lung parenchyma without COMP (A) and pericartilage airway region with COMP accumulation (B). (**C**) COMP protein (red) is located in fibrotic region of IPF Lungs. (D–G) Co-localization of COMP and vimentin in tissue obtained from IPF lung. The green fluorescence represents COMP and the red fluorescence shows fibroblasts marker; vimentin. Nuclei were counterstained with 4, 6- diamidino-2-phenylindole (blue). Yellow represents co-expression of COMP (green) and vimentin (red) in IPF lung as a yellow tinge. All figures shown are representatives of more than 3 experiments and magnification of 40×.

COMP was one of the most significantly increased genes in our previously published microarray data [17] and qRT-PCR confirmed its up-regulation in the same tissues (Figure 1A–B). We also verified the array result in a separate, larger cohort consisting of 115 IPF lung samples and 154 normal histology controls, by using nCounter® expression analysis and demonstrated that COMP mRNA was significantly increased in IPF lungs (8.8 Fold change, P- value = <0.05 compared to normal histology lungs) (Figure 1C). To compare COMP protein levels in IPF lungs to those in control lungs, we performed western blot analysis. We found a significant increase of COMP protein levels in IPF lungs (Figure 1D–E). In order to localize COMP in IPF lungs, we performed Immunohistochemistry (IHC) analysis. In normal histology lungs, COMP was mainly located in the cartilaginous areas of the large airways (Figure 2B). The IPF lung exhibited expression of COMP (red in color) in areas of dense fibrosis and myofibroblast foci (Figure 2C). To identify the types of cell that secrete COMP protein in the lungs, we performed immunofluorescence stains on frozen IPF and control lungs. COMP (green) and Vimentin (red) were co-localized in IPF lungs (Figure 2G) suggesting that COMP was secreted mainly by mesenchymal cells, most probably fibroblasts in IPF lungs.

### COMP is a TGF-β1 and Hypoxia Inducible Molecule

**Figure 3:**
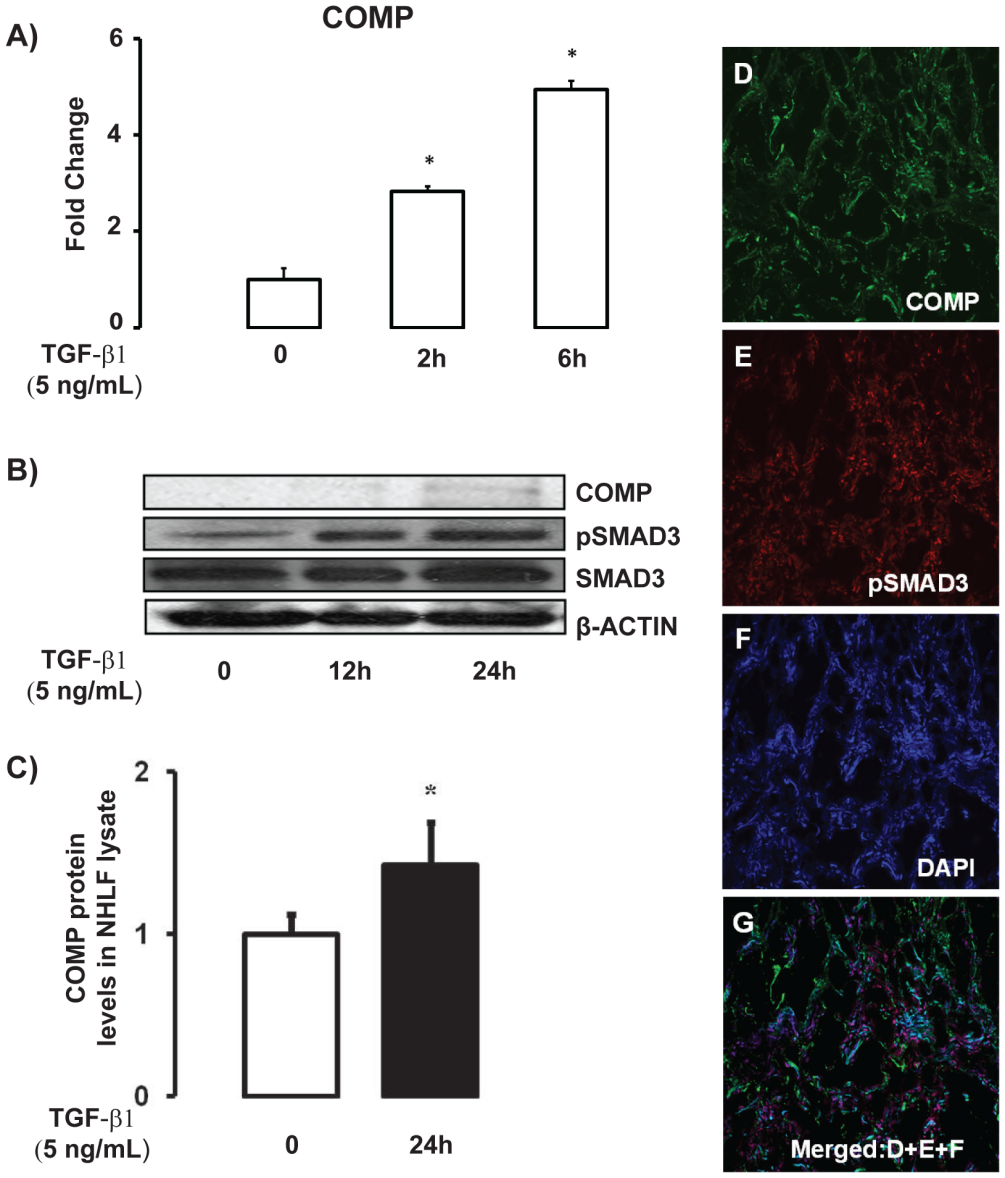
TGF-β1 induces COMP gene and protein expression. (A) COMP mRNA was determined by qRT-PCR in NHLF at 0, 2, and 6 hours after stimulation with recombinant TGF-β1 (5 ng/ml). (B) Western blots show an increase in COMP protein and pSMAD3 expression after 12–24 hours TGF-β1 treatment. For all western blot experiments β-actin was used as loading control and all figures shown are representatives of more than 3 experiments. (C) Primary fibroblasts were stimulated with TGF-β1 (5 ng/mL) for 24 hours and COMP protein was determined using ELISA assay. (D–G) COMP protein (green) is adjacent to the cells containing pSMAD3 in nuclei. The coexpression of nuclei (blue) and pSMAD3 (red) is observed in tissue obtained from IPF lung as purple color and it is surrounded with COMP (*green*) in the merged figure 3G.

After co-localizing COMP in fibroblasts of IPF lungs, we wanted to determine whether TGF-β1 regulates COMP expression. Stimulation of NHLF with TGF-β1 (5 ng/ml) induced increase in mRNA and protein levels of COMP in a time dependent manner as determined by qRT-PCR, western blot and ELISA (Figure 3A–C). To determine whether COMP expression in the fibrotic lungs could be associated with TGF-β1 effects, we co-localized phosphorylated SMAD3 (pSMAD3) and COMP. We found that COMP proteins were localized adjacent to cells containing pSMAD3 in the nuclei (Figure 3G) suggesting that this indeed was the case.

**Figure 4:**
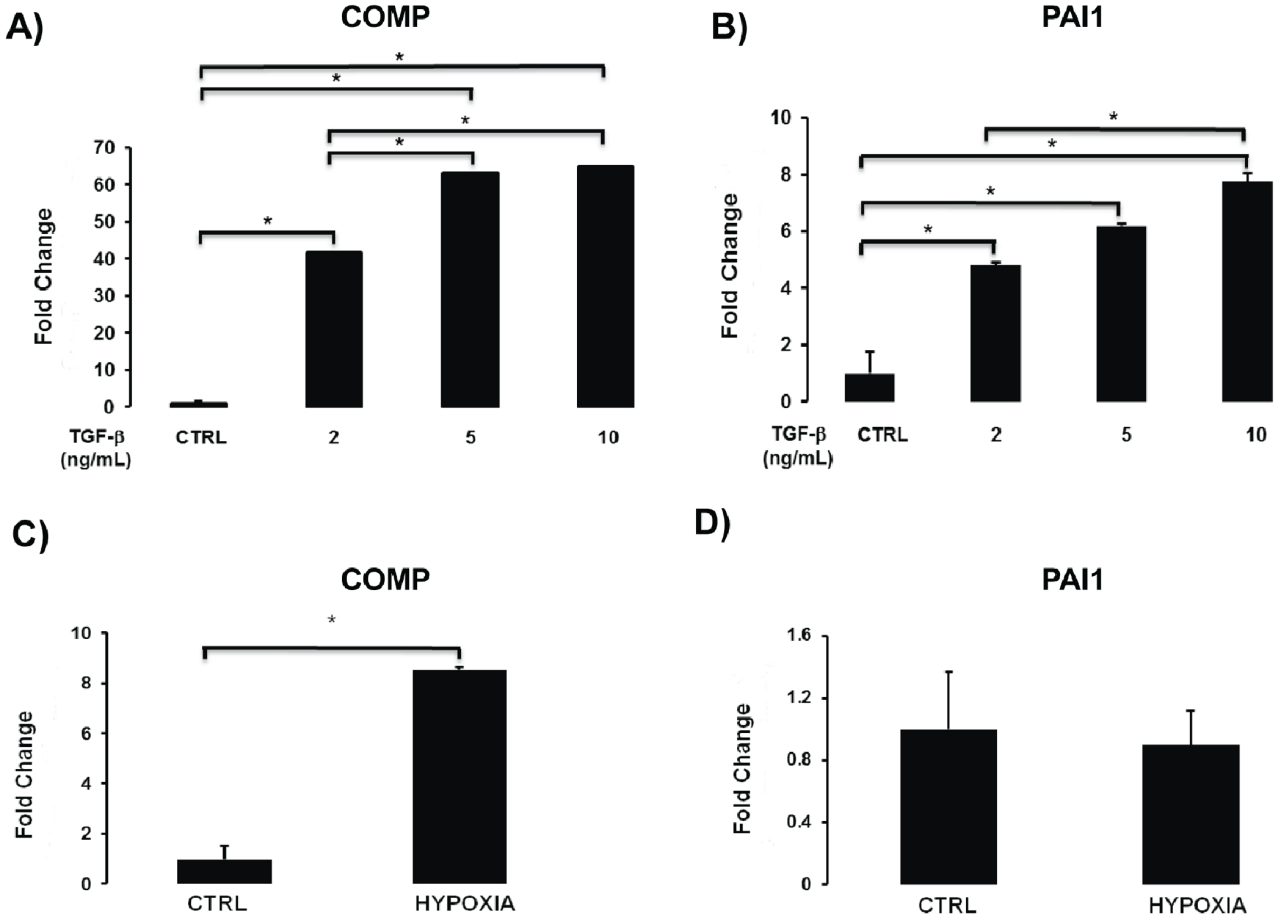
COMP induction by TGF β1 is dose dependent and Hypoxia induces COMP. A) COMP mRNA was determined by qRT-PCR in NHLF treated with 2, 5, and 10 ng/ml TGF β1. B) PAI induction by 2, 5, and 10 ng/ml TGF β1. C) Exposure of cells to extreme hypoxia (1% O_2_) for 24 hours causes induction of COMP mRNA (as measured by qRT-PCR), but not accompanied by a similar increase in PAI1 (D).

To determine the dose response of COMP induction by TGF-β1, we stimulated NHLF with 2, 5, and 10 ng/mL of TGF-β1. We observed increased expression of COMP with 2 ng/mL of TGF-β1, and an additional significant induction when we used a concentration of 5 ng/mL TGF-β1. Increasing the TGF-β1 concentration to 10 ng/mL had no additional effect on COMP expression (Figure 4A). In contrast, the TGF-β1 concentration of 10 ng/mL further increased the expression of PAI1, a highly TGF-β1 responsive gene (Figure 4B).

We also examined whether COMP was inducible by hypoxia. Exposure of NHLF cells to hypoxic conditions (1% O_2_) for 24 hours induced a significant increase COMP gene expression (Figure 4C) without evidence of an increase in TGF-β1 as can be observed from lack of increase in PAI1 (Figure 4D). This observation suggests that COMP may also be induced by extreme hypoxia conditions, potentially independent of TGF-β1.

### COMP Modulated TGF-β Signaling

**Figure 5:**
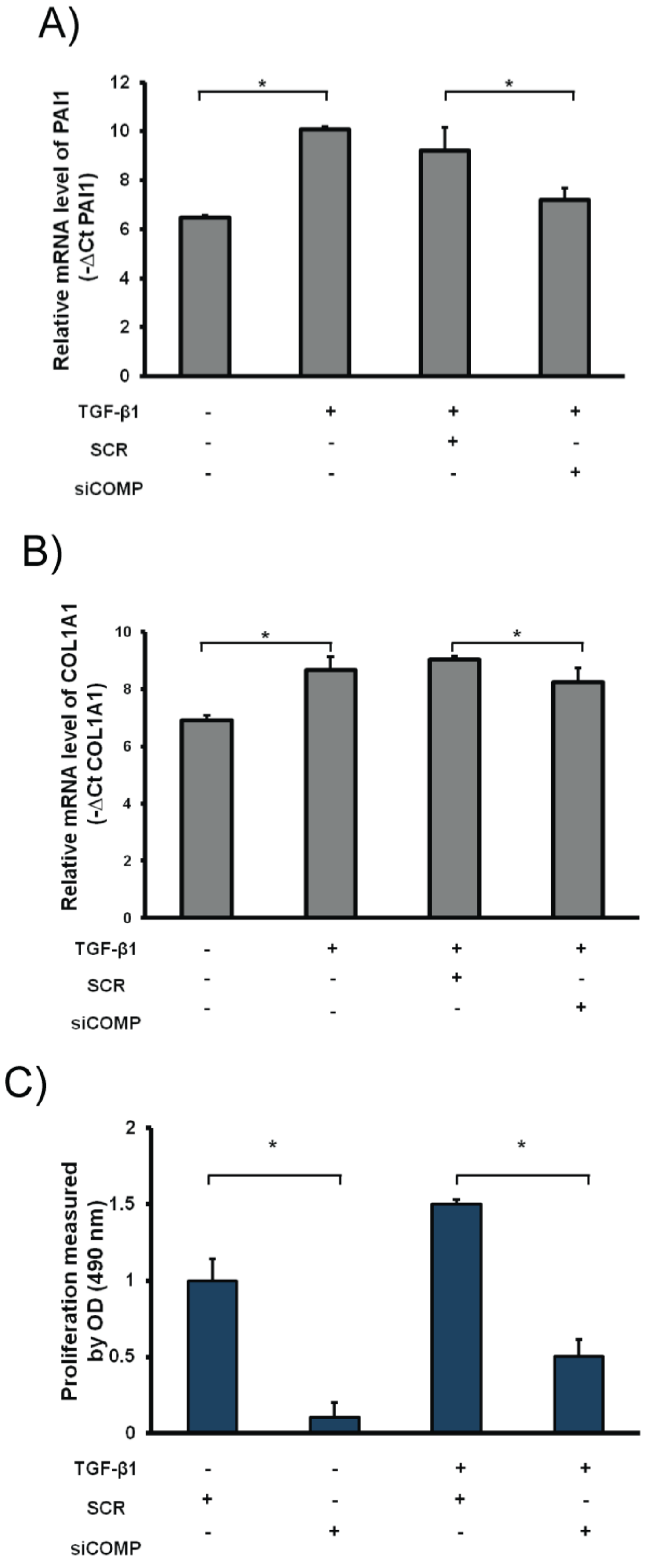
COMP modulates TGF-β signaling. NHLF were transfected with 70(SCR), treated for 6 hours with TGF-β1 (5 ng/mL) and RNA extracted after 24 hours. (A–B) qRT-PCR was used to determine mRNA levels of PAI1 and COL1A1 in NHLF. (C) The effect of COMP inhibition using siRNA on TGF-β1 induced NHLF proliferation.

In order to determine whether COMP plays a role in modulating TGF-β1 signaling, we measured mRNA levels of profibrotic and TGF-β responsive genes PAI1 and COL1A1. After silencing COMP, the expression of PAI1 and COL1A1 was significantly reduced (Figure 5 A–B). As it has been reported that TGF-β1 induced lung fibroblast proliferation [44], we examined NHLF proliferation rate after treating the cells with siCOMP and TGF-β1. The results showed that silencing COMP expression reduced TGF-β1 induced NHLF proliferation rate (Figure 5C) suggesting that indeed COMP had a role in modulating TGF-β1 signaling as previously described [19].

### Increased Concentration of COMP Protein in Serum of IPF Patients is Associated with Decline of FVC

**Figure 6:**
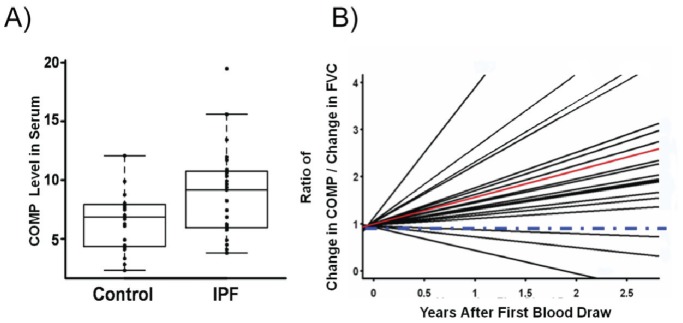
COMP Protein levels in serum of IPF patients are associated with decline of FVC. (A) ELISA assay was used to determine concentrations of COMP in serum of IPF patients (n = 20) compared to controls (n = 20) (t-test, P-value <0.05). (B) Data from patients (n = 23) showed the ratio of change in COMP and the decline of FVC in a longitudinal study. Red line represents average value of ratio of change in COMP level vs. change in FVC. The blue line represents baseline of COMP level in each participant whereas each serves as its own control.

We next compared concentration of COMP protein in the Serum of IPF patients (n = 20) and controls (n = 20). We used COMP ELISA Assay and determined that COMP concentrations were significantly increased in the serum of IPF patients when compared with controls (P-value = 0.004, mean = 9.977 and SD = 4.422 for IPF and mean = 6.475 and SD = 2.473 for controls (Figure 6A). To determine whether COMP changed with disease progression, we used samples from 23 patients who were prospectively followed up for 2.5 years with periodic blood draws and pulmonary function tests. We found that COMP protein levels increased in time dependent fashion and showed a significant correlation with the decline of force vital capacity in the majority of patients (Figure 6B).

## Discussion

In this study we investigated the levels and potential role of COMP in IPF. COMP is among the most up-regulated genes in IPF by microarrays, a result validated by both qRT-PCR and nCounter® expression system. COMP protein is localized to vimentin expressing cells in IPF lungs and is usually found adjacent to cells that have nuclear p-SMAD3. Stimulation of NHLF with TGF-β1 induces up-regulation of COMP gene expression and COMP protein levels. The silencing of COMP reduces PAI1 and COL1A1 gene expression and inhibits TGF-β1 induced fibroblast proliferation. COMP concentrations are increased in serum of IPF patients compared to control and the serum concentration of COMP in IPF patients continue to increase over time and correlate with disease progression as reflected by decline in FVC. Our observations highlight the potential role of COMP in IPF and support its’ proposed role of as a modulator of TGF-β1 signaling.

Our finding that COMP protein level is increased in IPF sera and IPF lungs is relevant in understanding the pathogenesis of IPF lungs. COMP mutations have been described as the cause of pseudoachondroplasia (PSACH) and multiple epiphyseal dysplasia (MED) [45]–[47]. The concentration of COMP is decreased in both serum and plasma of PSACH or MED patients [48], [49] which could be explained by accumulation of mutated COMP in granular or lamellar in endoplasmic reticulum (ER) of the chondrocytes [50], [51]. In contrary, COMP levels are increased in patients with rheumatoid arthritis (RA) and the molecule has been proposed as a potential biomarker for RA and osteoarthritis (OA) activity [25]. Similarly COMP is increased in scleroderma dermal fibroblasts and in serum of patients with systemic sclerosis [31]–[33] and also in cirrhotic livers [38]. While it has not been studied directly in the lung, a recent longitudinal study in patients with systemic scleroderma with lung involvement suggested that elevated COMP concentrations in serum were predictive of mortality [52]. While we did not assess mortality, we did show an increase in COMP proteins in the lungs and bloods of patients with IPF and demonstrated that the increase was associated with decline of FVC over time, concurrent with previous results in other disease and suggesting that COMP should be added to repertoire of proteins evaluated as potential peripheral blood biomarkers in IPF.

While we could not directly demonstrate COMP induction by TGF-β1 in human IPF lungs, we demonstrated that COMP mRNA and protein levels were increased after stimulation of human lung fibroblasts with TGF-β1. This is consistent with other reports that demonstrated induction of COMP after TGF-β1 stimulation in keloid and dermal fibroblast [30], [33]. Consistent with that, we have shown that COMP expression in the IPF lung is generally distributed around cells that express vimentin and that have abundant phosphorylated nuclear SMAD3, indicative of TGF-β1 stimulation. This is of particular interest because it had been described that COMP directly binds to members of TGF-β1 family and enhances their signal transduction activities [19]. When we examined the impacts of TGF-β1 on NHLF after the silencing of COMP, we found reduction in TGF-β1 target genes as well as significant reduction in TGF-β1 induced fibroblast proliferation, suggesting that indeed COMP does enhance TGF-β1 signaling. While it is difficult to establish experimentally a similar relation in the human IPF lung, our results that demonstrate co-localization of COMP to areas where there is evidence of TGF-β1 activities, add to these observations and suggest the possibility of a positive feedback loop between COMP and TGF-β1 activities in the IPF lung.

The fibrotic lung is characterized by the intensive accumulation of extracellular matrix (ECM). The pathologic ECM depositions in fibrotic lungs include collagens (I, III, V, VI and VII), fibronectin, elastin, and cartilage related proteins [53]. Interestingly, we found COMP was increased in dense fibrotic areas of lung parenchyma in IPF. We also localized COMP in normal lung but only around cartilage possessing airway (Figure 2A–B). In the case of COMP expression in chronic diseases such as pseudoachondroplasia (PSACH) and multiple epiphyseal dysplasia (MED), rheumatoid arthritis, systemic sclerosis and pathological would healing (Keloid), it was reported that COMP inhibits normal collagen fibrils formation and destabilize normal extracellular matrix formation in areas where COMP molecules are excessively higher in the relationship to collagen [30], [54]. It was suggested that this inhibition cause increased lung rigidity by the lost elasticity typical of normal ECM. This may be of particular interest, because of the evidence that abnormal ECM rigidity plays a significant role in the pathogenesis of fibrosis [55]. In this context, it is of interest to note that other cartilage related proteins such as osteopontin [10], [56], periostin [57], and YKL-40 [58] have been described as being significantly increased in IPF.

It is critically important to acknowledge few limitations of our study. First, we provide evidence on COMP regulation through TGF-β1 and its involvement in TGF-β1 signaling cascade in NHLF, but we do not provide similar data in-vivo. We feel that the co-localization of vimentin, pSMAD3 and COMP in fibrotic foci in human IPF lungs suggests that TGF-β1 induces COMP secretion mostly in fibrotic regions of IPF lungs and to some extent more informative than using a limited model of lung fibrosis. To that extent we did observe that in-vitro exposing NHLF to extreme hypoxia *in-vitro* did also induce COMP, but it is unclear whether this mechanism would be relevant *in-vivo*. Clinically, our findings are also limited, while we offer compelling evidence of the relationship between COMP protein levels in serum and FVC, we are aware that the numbers are limited and we did not capture patients for this study in earlier stages of the disease. Our center is a tertiary medical facility, which gets regional and national referrals, and thus the populations of the study were at later stages of the disease. Despite this shortcoming, we were able to show the continuous increase of serum COMP level in 2.5 years. Considering its role in regulation of matrix rigidity, the observation that elevated COMP concentrations in serum of IPF patients are associated with a decline of FVC provides support to our hypothesis that COMP plays a role in pathogenesis of IPF and should be further evaluated as a biomarker for disease activity in IPF.

In this manuscript we demonstrate that the mRNA and protein expression levels of COMP, an extracellular matrix protein that accentuates TGF-β1 signaling and is associated with extracellular matrix polymerization and stiffness, are high in IPF lungs compared to controls. COMP serum protein concentrations are increased in IPF patients and correlate with the decline of FVC over time in individuals with IPF. We also demonstrate data that COMP may be induced by TGF-β1 in the IPF lung and that at least *in-vitro* serves as an enhancer of TGFβ1 signaling as previously proposed. Taken together, our results should encourage more research into the potential use of COMP as a biomarker for disease activity and TGF-β1 activity in patients with IPF. Hence, studies that explore modalities that affect COMP expression, alleviate extracellular matrix rigidity and lung restriction in IPF and interfere with the amplification of TGF-β1 signaling should be persuaded.
